# Ambient Temperature and Early Delivery of Singleton Pregnancies

**DOI:** 10.1289/EHP97

**Published:** 2016-08-31

**Authors:** Sandie Ha, Danping Liu, Yeyi Zhu, Sung Soo Kim, Seth Sherman, Pauline Mendola

**Affiliations:** 1 Epidemiology Branch, and; 2 Biostatistics and Bioinformatics Branch, Division of Intramural Population Health Research, *Eunice Kennedy Shriver* National Institute of Child Health and Human Development, Rockville, Maryland, USA; 3 Emmes Corporation, Rockville, Maryland, USA

## Abstract

**Background:**

Extreme temperature is associated with adverse birth outcomes but it is unclear whether it increases early delivery risk.

**Objectives:**

We aimed to determine the association between ambient temperature and early delivery.

**Methods:**

Medical records from 223,375 singleton deliveries from 12 U.S. sites were linked to local ambient temperature. Exposure to hot (> 90th percentile) or cold (< 10th percentile) using site-specific and window-specific temperature distributions were defined for 3-months preconception, 7-week periods during the first two trimesters, 1 week preceding delivery, and whole pregnancy. Poisson regression with generalized estimating equations calculated the relative risk (RR) and 95% confidence interval for early deliveries associated with hot/cold exposures, adjusting for conception month, humidity, site, sex, maternal demographics, parity, insurance, prepregnancy body mass index, pregnancy complications, and smoking or drinking during pregnancy. Acute temperature associations were estimated separately for warm (May–September) and cold season (October–April) in a case-crossover analysis using conditional logistic regression.

**Results:**

Compared with mild temperature (10–90th percentile), exposure to hot or cold during weeks 1–7 increased risk for early preterm (< 34 weeks) [RR_hot_: 1.11 (95% CI: 1.01, 1.21); RR_cold_: 1.20 (95% CI: 1.11, 1.30)], late preterm (34–36 weeks) [RR_cold_: 1.09 (95% CI: 1.04, 1.15)], and early term (37–38 weeks) [RR_hot_: 1.04 (95% CI: 1.02, 1.07); RR_cold_: 1.03 (95% CI: 1.00, 1.05)] delivery. Findings were similar for hot exposures during weeks 15–21. Examining deliveries at each week from 23 through 38, whole-pregnancy hot exposures increased delivery risk by 6–21% at weeks 34 and 36–38. In the case-crossover analysis, a 5°F increase during the week preceding delivery was associated with 12–16% higher and 4–5% lower early delivery risk during warm and cold season, respectively.

**Conclusions:**

Both acute and chronic ambient temperature extremes may affect early delivery risk.

**Citation:**

Ha S, Liu D, Zhu Y, Kim SS, Sherman S, Mendola P. 2017. Ambient temperature and early delivery of singleton pregnancies. Environ Health Perspect 125:453–459; http://dx.doi.org/10.1289/EHP97Citation: Ha S, Liu D, Zhu Y, Kim SS, Sherman S, Mendola P. 2017. Ambient temperature and early delivery of singleton pregnancies. Environ Health Perspect 125:453–459; http://dx.doi.org/10.1289/EHP97

## Introduction

Preterm birth is typically defined as birth of an infant at < 37 weeks of gestation. In the United States, the prevalence of preterm birth in 2013 was 11.4%, but the prevalence varies depending on the characteristics of the population in question (Martin et al. 2015). Although the prevalence of preterm birth has been declining since 2006 in the United States (Martin et al. 2015), it is still increasing in most countries with reliable trend data (Blencowe et al. 2013). Preterm infants are highly vulnerable to adverse outcomes including infant mortality; complications related to respiratory function, neurodevelopment, behavioral development; and many other sequelae during childhood as well as later in life (Saigal and Doyle 2008).

The effects of prenatal exposure to environmental factors on adverse birth outcomes including preterm birth have received increasing attention in the literature (Nieuwenhuijsen et al. 2013; Shah et al. 2011). With worldwide concern regarding global warming and the expected increase in the frequency and severity of extreme weather events (Luber and McGeehin 2008), health effects of extreme ambient temperature during pregnancy are of great public health interest. The potential association with preterm birth is particularly important given the high prevalence and deleterious consequences.

Although the exact mechanisms remain to be elucidated, the association of temperature extremes with preterm birth is biologically plausible. One potential pathway could be that stress associated with exposure to temperature extremes might trigger early labor (Dreiling et al. 1991; Simčič et al. 2015; Stan et al. 2013). Despite biological plausibility, studies on the potential effects of extreme ambient temperature during pregnancy are limited and inconsistent (Carolan-Olah and Frankowska 2014). These discrepancies are likely related to heterogeneity in study attributes such as design and analysis, geographic location, population, method of exposure assessment, windows of exposure under consideration, and/or method of assessment for preterm birth (Carolan-Olah and Frankowska 2014). Another issue is related to regional adaptation. It is likely that deviation from the usual environment is what drives temperature-related risk, and populations typically adapt to the usual climatic condition in their region (Guo et al. 2014; Yang et al. 2015). For example, individuals from cooler regions may experience heat stress at a lower temperature than individuals living in hotter regions. In addition, many existing studies on temperature and preterm birth have been generally more interested in acute effects of heat, leaving the potential effects of cold temperature and that of chronic exposures relatively understudied (Arroyo et al. 2015; Auger et al. 2014; Wang et al. 2013). Last, few studies have investigated the risk of early delivery associated with temperature during the week preceding delivery, and only one used case-crossover design where a woman serves as her own control (Basu et al. 2010).

The objective of this paper was to determine the association between extreme ambient temperature at several potentially critical time windows during pregnancy and early delivery (preterm birth and early term birth) in a contemporary U.S. obstetric cohort. We assessed both temperature extremes (cold and hot) using site-specific distribution of temperature to adjust for regional acclimation.

## Methods

### Study Population

**Figure 1:**
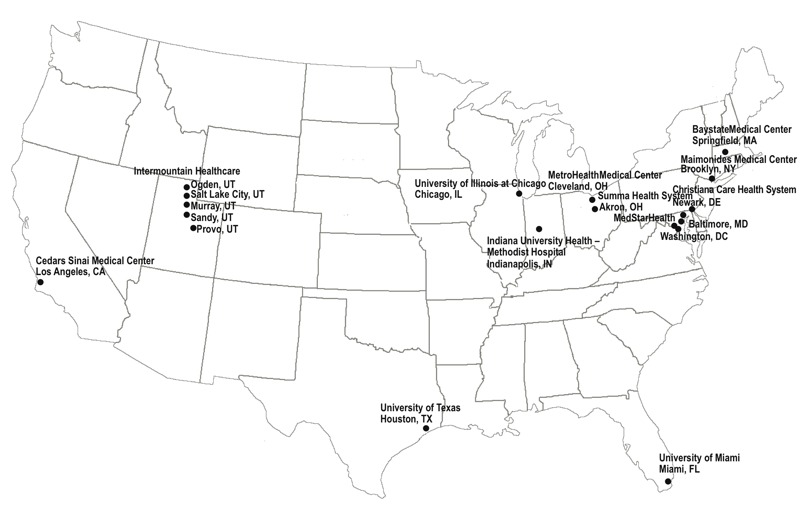
Spatial distribution of study sites. Reprinted from *Journal of Allergy and Clinical Immunology*, 138/2, Mendola P, Wallace M, Hwang SH, Liu D, Robledo C, Männistö T, Sundaram R, Sherman S, Ying Q, Grantz KL, Preterm birth and air pollution: Critical windows of exposure for women with asthma, Pages 432–440e5, Copyright (2016), with permission from Elsevier.

Data came from the Air Quality and Reproductive Health study, which linked local meteorological data to participants in the Consortium on Safe Labor (CSL; https://dash.nichd.nih.gov/Study/Study?id=2331). CSL was an observational cohort study which included 228,438 deliveries at ≥ 23 weeks of gestation from 12 clinical centers (15 hospital referral regions) across the United States from 2002 through 2008 (Figure 1). Data on maternal demographics; medical history; and labor and delivery, obstetric, and neonatal outcomes were extracted from electronic delivery records and supplemented with *International Classification of Diseases*, *9th Revision* (ICD-9) codes in the hospital discharge summaries. A detailed description of CSL data and validation studies has been previously published (Zhang et al. 2010). The study was approved by the institutional review boards of all participating institutions listed in the acknowledgement section. Informed consent was not required because the study was based on anonymous data. After excluding multiple births (*n* = 5,053), and those without exposure information (had exposure windows occurring before 2001 when exposure assessment period started, *n* = 10), 223,375 singleton births remained in the study sample.

### Exposure Assessment

Hourly temperature data were obtained from the Weather Research and Forecasting (WRF; http://www.wrf-model.org/index.php) model v3.2.1 and were linked based on data collected in the delivery hospital referral region for each woman. A description of the WRF modeling approach and its performance has been previously reported (Zhang et al. 2014). Due to the anonymity of the CSL data, we were unable to obtain residential addresses for detailed spatial interpolation of exposure. Thus, we used the 15 distinct non-overlapping hospital referral regions (415–312,644 km^2^) as a proxy for maternal residence and local mobility (e.g., frequent short-range spatial movements related to daily activities such as work, errands).

Despite efforts in understanding the role of environmental exposures on preterm birth, the etiologically critical exposure window for temperature to affect preterm risk is unclear. We explored several windows except for the third trimester because a considerable number of preterm births ended before the third trimester (*n* = 2,366, 9.1%). Exposures were assessed using average daily temperature over 3 months preconception [91 days before estimated last menstrual period (eLMP)]; weeks 1–7, 8–14, 15–21 and 22–28; and the whole pregnancy period (eLMP through date of delivery). eLMP was back-calculated from date of delivery using the best gestational age estimate in delivery records. The 7-week windows for the first two trimesters were chosen because they best captured the change in risk associated with environmental exposures over time in our data (Mendola et al. 2016). To reflect regional acclimation, we categorized our temperature exposure using local temperature distributions among study participants for each pregnancy window. For each site and separately for each pregnancy window, we created the temperature distribution, then defined exposures based on the following cut-offs: cold (< 10th percentile), hot (> 90th percentile), and mild (10–90th percentile).

To assess acute exposure, we compared the average temperature during the week preceding delivery for each subject with an early delivery with two control periods for the same subject in a case-crossover analysis. The week preceding delivery was designated as the hazard period because literature on acute health effects of ambient temperature on other delivery outcome shows meaningful associations within this time window (Basu et al. 2010; Schifano et al. 2013). We used a symmetric bidirectional method to select the control periods: the second week after delivery, and the week 2 weeks before delivery (Bateson and Schwartz 1999). The temperature difference between a hazard period and control period was expected to be small; therefore, we included temperature as a continuous exposure to retain more information on the temperature change.

### Outcome and Covariates

All outcome and covariates information was obtained from electronic medical records and ICD-9 codes in the hospital discharge summaries. The main outcome of this study was early delivery, which was defined using best clinical gestational age in the delivery records. We categorized early deliveries into several mutually exclusive groups (Spong 2013)—early preterm births (< 34 weeks), late preterm births (34–36 weeks), early term births (37–38 weeks)—and compared them with full-term births (≥ 39 weeks). Covariates included maternal age, race, infant sex, marital status, prepregnancy body mass index (BMI), parity, gestational complications, smoking or alcohol use during pregnancy, insurance status, month of conception, humidity, and study site. We considered calendar year as a potential covariate but it was not related to exposure in our data. Nominal variables where appropriate were dummy coded.

### Statistical Analyses

We determined associations between ambient temperature and early delivery using two different methods. First, we employed full cohort analyses with Poisson regression to determine the relative risk (RR) and 95% confidence intervals (CI) of early preterm birth, late preterm birth, and early term birth associated with extreme temperature exposures using the full-term group as reference. This was done by comparing the cold/hot groups with the mild group for different pregnancy windows, adjusting for covariates previously described. A total of 19,210 (8.6%) women had more than one singleton delivery during the study period, so we used robust standard errors from generalized estimating equations to adjust for the clustering effects.

Because the length of pregnancy was different for early deliveries and full-term births, we did not directly compare whole-pregnancy exposures. Instead, we compared whole-pregnancy exposure of early deliveries at a given week (range, weeks 23–38) with that of ongoing pregnancies truncated to same length of gestation. For example, whole-pregnancy average temperatures for deliveries at week 32 were compared with all ongoing pregnancies using temperature exposures up to week 32. For these analyses, the cut-offs used to define cold/hot extremes were specific to site and the distribution of temperature was based on pregnancies at risk for delivery each week (i.e., deliveries before the index week of the analysis were not included). Fitting regression models stratified by week of delivery is similar to a pregnancy-at-risk approach. The risk estimate can be interpreted as the risk of delivery during a given week associated with whole-pregnancy exposures up to that week.

Second, we conducted a case-crossover analysis to examine the acute association between exposure during the week preceding delivery and early delivery. This design allowed us to control for observed or unobserved subject-level characteristics by comparing a hazard period with alternate control periods, where each woman served as her own control. Conditional logistic regression with robust standard error was used to estimate the odds of early delivery for each 5°F increase in temperature after adjustment for relative humidity. Analyses were stratified for early deliveries during warm (May–September) and cold (October–April) seasons. We restricted this analysis to only the first early delivery case for each woman in the cohort (87,832 of 92,710 total early delivery cases, or 41.5% of the whole cohort).

### Sensitivity Analyses

We performed several sensitivity analyses to ensure the robustness of our findings. These analyses involved *a*) restricting analyses to only spontaneous preterm births; *b*) additionally adjusting for exposures to two common air pollutants during the same window: ozone and particulate matter with diameter < 2.5 μm, which were estimated using modified Community Multiscale Air Quality models based on emissions, meteorology, photochemical properties of pollutants, and population density, and fused to monitor data using inverse distance weighting (Chen et al. 2014); and *c*) restricting analyses to nulliparous women to ensure no residual confounding by previous early delivery. We also stratified our analyses by several factors including insurance status (private, nonprivate), site (12 sites), maternal age (< 35, ≥ 35 years), gestational diabetes (yes, no), hypertensive disorders of pregnancy (yes, no), and maternal obesity (yes, no).

## Results

Table 1 describes the characteristics of the study participants by early delivery status. Among the 223,375 singleton births included in the study, 8,767 (3.9%) were early preterm, 17,363 (7.8%) were late preterm, 66,580 (29.8%) were early term, and 130,665 (58.5%) were full term. Early deliveries were more frequent among male infants, mothers who were black, < 20 or > 35 years of age, not married, smoked or consumed alcohol during pregnancy, had pregnancy complications, had public insurance, or conceived during the winter months. Early deliveries were also less frequent among women who were normal weight. Table S1 describes the distribution of temperature categories by pregnancy windows and early delivery status. The site-specific absolute temperature distributions that were used to define hot/cold/mild exposure categories are also reported by site and pregnancy windows in Table S2. The temperature cut-off used to define cold during each pregnancy window was much lower in colder areas than in warmer areas. For example, cold during the pre-pregnancy period was defined as < 27.4°F in Massachusetts (Baystate Medical Center) but < 70.1°F in Florida (University of Miami).

**Table 1:** Characteristic of study population by early delivery status (*n* = 223,375).

Characteristics	Early preterm birth (< 34 weeks) (*n *= 8,767)		Late preterm birth (34–36 weeks) (*n* = 17,363)		Early term birth (37–38 weeks) (*n *= 66,580)		Full-term birth (≥ 39 weeks) (*n *= 130,665)
	*n* (%)	*p*-Value^*a*^	*n* (%)	*p*-Value^*a*^	*n* (%)	*p*-Value^*a*^	*n* (%)
Race/ethnicity		< 0.0001		< 0.0001		< 0.0001
Non-Hispanic white	2,944 (33.6)		7,640 (44.0)		32,525 (48.9)		67,432 (51.6)
Non-Hispanic black	3,332 (38.0)		5,128 (29.5)		15,440 (23.2)		26,355 (20.2)
Hispanic	1,551 (17.7)		3,023 (17.4)		11,445 (17.2)		22,792 (17.4)
Asian/Pacific Islander	200 (2.3)		571 (3.3)		3,079 (4.6)		5,325 (4.1)
Other	311 (3.6)		414 (2.4)		1,507 (2.3)		2,998 (2.3)
Unknown	429 (4.9)		587 (3.4)		2,584 (3.9)		5,763 (4.4)
Maternal age (years)		0.4449		0.8628		< 0.0001
< 20	1,124 (12.8)		1,895 (10.9)		5,669 (8.5)		12,007 (9.2)
20–24	2,243 (25.6)		4,398 (25.3)		16,047 (24.1)		33,893 (25.9)
25–29	1,992 (22.7)		4,536 (26.1)		18,614 (28.0)		37,070 (28.4)
30–34	1,831 (20.9)		3,714 (21.4)		15,519 (23.3)		29,081 (22.3)
≥ 35	1,556 (17.8)		2,804 (16.2)		10,638 (16.0)		18,437 (14.1)
Unknown	21 (0.2)		16 (0.1)		93 (0.1)		177 (0.1)
Infant sex		< 0.0001		< 0.0001		< 0.0001
Female	4,083 (46.6)		8,152 (47.0)		32,050 (48.1)		64,643 (49.5)
Male	4,524 (51.6)		9,138 (52.6)		34,397 (51.7)		65,873 (50.4)
Unknown	160 (1.8)		73 (0.4)		133 (0.2)		149 (0.1)
Marital status		< 0.0001		< 0.0001		0.1187
Not married	4,562 (52.0)		7,854 (45.2)		24,746 (37.2)		47,832 (36.6)
Married	3,802 (43.4)		8,927 (51.4)		39,615 (59.5)		78,831 (60.3)
Unknown	403 (4.6)		582 (3.4)		2,219 (3.3)		4,002 (3.1)
Parity		0.6779		< 0.0001		< 0.0001
0	3,852 (43.9)		6,641 (38.3)		23,311 (35.0)		55,220 (42.3)
1	2,253 (25.7)		4,933 (28.4)		22,130 (33.2)		39,073 (29.9)
≥ 2	2,662 (30.4)		5,789 (33.3)		21,139 (31.8)		36,372 (27.8)
Prepregnancy BMI		< 0.0001		< 0.0001		< 0.0001
< 18.5	282 (3.2)		702 (4.0)		2,597 (3.9)		4,395 (3.4)
18.5–24.9	2,195 (25.0)		5,577 (32.1)		23,087 (34.7)		48,182 (36.9)
25–29.9	1,156 (13.2)		2,528 (14.6)		9,826 (14.8)		19,985 (15.3)
≥ 30	1,235 (14.1)		2,431 (14.0)		8,590 (12.9)		15,619 (12.0)
Unknown	3,899 (44.5)		6,125 (35.3)		22,480 (33.8)		42,484 (32.5)
Smoking during pregnancy	1,002 (11.4)	< 0.0001	1,665 (9.6)	< 0.0001	4,454 (6.7)	< 0.0001	7,809 (6.0)
Alcohol use during pregnancy	284 (3.2)	< 0.0001	384 (2.2)	< 0.0001	1,222 (1.8)	0.0155	2,200 (1.7)
Hypertensive disorders of pregnancy	1,675 (19.1)	< 0.0001	2,501 (14.4)	< 0.0001	4,551 (6.8)	< 0.0001	4,317 (3.3)
Gestational diabetes	533 (6.1)	< 0.0001	1,325 (7.6)	< 0.0001	4,548 (6.8)	< 0.0001	4,934 (3.8)
Insurance type		< 0.0001		< 0.0001		0.3368
Private	3,863 (44.1)		8,592 (49.5)		37,760 (56.7)		74,688 (57.2)
Public	3,930 (44.8)		6,760 (38.9)		21,563 (32.4)		39,894 (30.5)
Other	166 (1.9)		294 (9.9)		875 (1.3)		1,647 (1.3)
Unknown	808 (9.2)		1,717 (9.9)		6,382 (9.6)		14,436 (11.1)
Month of conception		< 0.0001		0.0016		0.5574
January	770 (8.8)		1,488 (8.6)		4,953 (7.4)		8,956 (6.9)
February	678 (7.7)		1,119 (6.4)		4,182 (6.3)		8,185 (6.3)
March	713 (8.1)		1,218 (7.0)		4,782 (7.2)		9,349 (7.2)
April	634 (7.2)		1,277 (7.4)		5,090 (7.6)		10,573 (8.1)
May	668 (7.6)		1,431 (8.2)		5,823 (8.8)		11,310 (8.7)
June	676 (7.7)		1,472 (8.5)		5,503 (8.3)		11,019 (8.4)
July	764 (8.7)		1,577 (9.1)		5,634 (8.5)		11,302 (8.7)
August	726 (8.3)		1,502 (8.7)		5,969 (9.0)		11,520 (8.8)
September	677 (7.7)		1,482 (8.5)		5,767 (8.7)		11,524 (8.8)
October	807 (9.2)		1,513 (8.7)		6,131 (9.2)		12,425 (9.5)
November	819 (9.3)		1,576 (9.1)		6,209 (9.3)		12,329 (9.4)
December	835 (9.5)		1,708 (9.8)		6,537 (9.8)		12,173 (9.3)
^***a***^*p*-Values were obtained by generalized estimating equations, accounting for multiple pregnancies of the same woman during the study period.							

**Figure 2:**
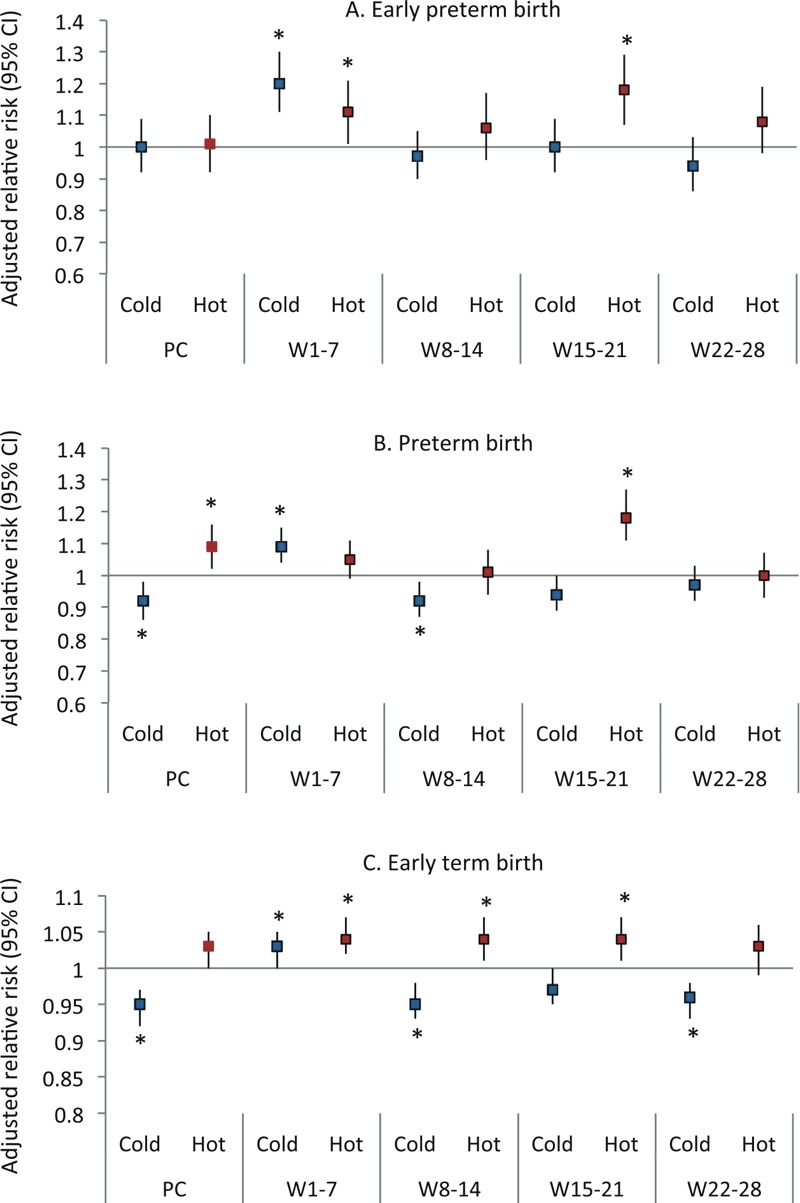
Adjusted relative risk of preterm birth associated with extreme ambient temperature by pregnancy windows. Models were adjusted for all covariates in Table 1, humidity, and study site. *Statistical significance at alpha < 0.05. Abbreviations: PC, preconception; W, week.

Unadjusted and adjusted associations between extreme ambient temperatures and early deliveries were similar, so we presented the adjusted associations in Figure 2 and Table S3. After adjustment for covariates, compared with mild temperature, cold exposures during weeks 1–7 were associated with a 20% (95% CI: 11%, 30%), 9% (95% CI: 4%, 15%), and 3% (95% CI: 0%, 5%) higher risk of being early preterm, late preterm, and early term, respectively. However, exposures during preconception and other prenatal windows appeared to be inversely associated with early delivery risk.

Hot exposures during weeks 1–7 were associated with an 11% (95% CI: 1%, 21%), and 4% (95% CI: 2%, 7%) increased risk of early preterm and early term, respectively. Similar findings were also observed for hot exposures during weeks 15–21 with an increased risk of 18% for early preterm (95% CI: 7%, 29%) and late preterm (95% CI: 11%, 27%), and 4% (95% CI: 1%, 7%) for early term births (Figure 2; see also Table S3). Hot exposures during the preconception period were associated with a 9% (95% CI: 2%, 16%) increased risk of late preterm birth; and exposures during weeks 8–14 increased the risk of early term birth by 4% (95% CI: 1%, 7%). No significant associations were observed for any other window–outcome combination. When restricting the analysis to only spontaneous preterm births, the results remained consistent (see Table S4). When stratified by study site, the results were also generally consistent but confidence intervals became wider due to the lower sample size at each site (see Table S5). For example, in Massachusetts (Baystate Medical Center), the extreme hot cut-off for weeks 1–7 exposure was relatively low compared with other areas (67.4°F), but we still observed a 37% increase in risk of very preterm delivery (RR = 1.37; 95% CI: 0.98, 1.92). In some sites and exposure windows, the relationships were not consistent. For example, in Miami, where the cold cut-off was relatively high (69.9°F), the main effect of extreme cold at weeks 1–7 was lower than in the overall sample (see Table S5). Other sensitivity analyses also showed consistent findings (not shown).

**Figure 3:**
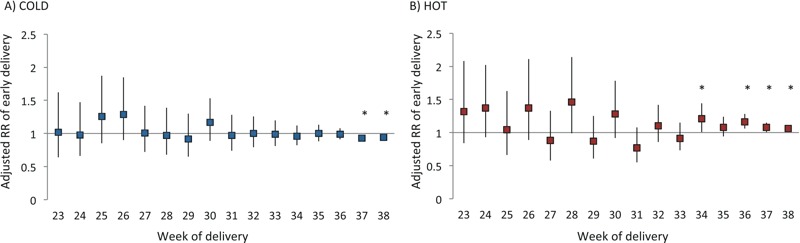
Adjusted relative risk of early delivery associated with average whole-pregnancy cold (*A*) and hot (*B*) by week of delivery. Whole-pregnancy exposure of deliveries during each week was compared with whole-pregnancy exposure truncated up to that week for ongoing pregnancies. Models were adjusted for all covariates in Table 1, humidity, and study site. *Statistical significance at alpha < 0.05.

Figure 3 describes the associations between whole-pregnancy exposures to extreme temperatures and early delivery. The risk estimates are also presented in Table S6. Whole-pregnancy averages were truncated for ongoing pregnancies up to a given week. In general, chronic exposures to hot extreme during the whole pregnancy up to the week of delivery were positively associated with early deliveries at weeks 34 and 36 to 38, whereas cold exposures were inversely associated with risk at weeks 37 and 38. No association was observed for any other weeks.

Our case-crossover analysis included a total of 87,832 first births that were delivered before 39 weeks of gestation. Results suggested a 12% to 16% increase in the odds of early delivery for 5°F increase (~ 2.8°C) in ambient temperature during the week preceding delivery in the warm season (Table 2). During the cold season, 5°F increase in ambient temperature during the week preceding delivery was associated with 4–5% decrease in risk, suggesting potential adverse effect with colder temperature.

**Table 2:** Adjusted odds of preterm birth associated with temperature during the week preceding delivery in the case-crossover analysis (*n* = 87,832).

Season	Early preterm birth (< 34 weeks)		Late preterm birth (34–36 weeks)		Early term birth (37–38 weeks)	
	*n*	OR^*a*^ (95% CI)	*n*	OR^*a*^ (95% CI)	*n*	OR^*a*^ (95% CI)
Cold	4,510	0.95 (0.93, 0.97)*	8,913	0.96 (0.95, 0.98)*	34,267	0.96 (0.96, 0.97)*
Warm	3,855	1.16 (1.12, 1.19)*	7,345	1.12 (1.10, 1.15)*	28,942	1.13 (1.12, 1.14)*
^***a***^ORs are for 5°F increase in temperature, and model was adjusted for relative humidity. *Statistical significance at alpha < 0.05.						

## Discussion

In this large multicenter cohort, exposures to both temperature extremes during early pregnancy (weeks 1–7) appeared to have a positive association with early preterm birth and early term birth, whereas only cold temperature had positive association with late preterm birth during this window. Hot temperature relative to usual environment also had positive associations with early delivery during various other windows during first two trimesters, whereas cold temperature had inverse associations. Acute exposures to both low and high temperature during the week preceding delivery increased the risk of early delivery during the cold and warm season, respectively. Examining deliveries at each week from week 23 through 38, we also found significant association between chronic whole-pregnancy hot (positive) and cold (inverse) exposures and risk of early delivery. Together, these results suggest that extreme ambient temperature may have early, acute, as well as chronic effects on early delivery.

The observed risks associated with both cold and hot exposure during weeks 1–7 are novel. Early pregnancy is generally a sensitive period to environmental hazards, and the developing fetus is highly susceptible to the oxidative stress and inflammatory effects that can be induced by heat/cold stress (Dreiling et al. 1991; Simčič et al. 2015). These responses can disturb trophoblast invasion, the pituitary–adrenocortico–placental system, and uterine blood flow, all of which may ultimately lead to preterm birth (Al-Gubory et al. 2010). The early association with temperature suggests that there may be an impact on placental development, which is further supported by the consistent findings when restricting to spontaneous preterm births. To address potential confounding across time windows, where women in the “hot” category for an early window might not be “hot” in a later window due to seasonal shifts, we included all shorter pregnancy time windows in a single model and the individual window results remained robust. Because prior research has been generally interested in the effects of recent/acute exposures on preterm birth, early pregnancy effects were rarely investigated. However, our findings suggested that this window is potentially important and warrants further investigation.

Contrary to our findings, a cohort study in Germany investigated temperature during first month and first trimester but found no evidence of an association with preterm birth (Wolf and Armstrong 2012). The discrepancy in findings may be partially explained by the larger geographical coverage and greater temperature range in our study. In addition, the referenced study was ecologic in nature and the authors were unable to adjust for many important confounders such as maternal race, gestational complications, prepregnancy BMI, and smoking/drinking during pregnancy. Our study also had a different reference group (≥ 39 weeks) compared with the referenced study, which used births ≥ 37 weeks according to the classic definition of preterm birth (WHO 2015).

Chronic whole-pregnancy effects have also received little attention (Carolan-Olah and Frankowska 2014; Strand et al. 2011). Our study is among the few in the United States to observe whole-pregnancy effects of temperature on early delivery. Kloog et al. examined whole-pregnancy average ambient temperature and preterm births in Boston, Massachusetts, and found that an interquartile (2.7°C) increase was associated with a 2% increase (95% CI: 0, 5%) in odds (Kloog et al. 2015). Our findings are also consistent with studies from other parts of the world. For example, studies from Uppsala, Sweden, and Guangzhou, China, reported significant associations between whole-pregnancy exposures to both temperature extremes and preterm birth (Bruckner et al. 2014; He et al. 2016). We found significant associations only for deliveries in later weeks. This may be attributable to lack of power because the number of early delivery cases in earlier weeks was much smaller. Nevertheless, the present findings may suggest that chronic stress potentially induced by higher temperature relative to usual environment may have important implications for pregnant women.

The acute associations observed in our case-crossover analysis were consistent with that from existing studies. We are aware of one case-crossover study among California births from 1999 through 2006, which showed that a 10°F increase in temperature during the week preceding delivery was associated with an 8.6% higher risk (Basu et al. 2010). Other studies around the world using different study designs also reported similar acute associations with exposure during the week preceding delivery (Arroyo et al. 2015; Auger et al. 2014; Schifano et al. 2013; Wang et al. 2013). Although cold exposure has received relatively less attention, a recent study by He et al. (2016) found that extreme cold, defined by the first percentile (< 7.6°C), during the 4 weeks before delivery was associated with approximately 18% increase in risk of premature birth in Guangzhou, China. These authors also found that the association was stronger for earlier preterm births, an observation also consistent with our results in the cohort analysis.

The reason for the inconsistent association between cold and early deliveries across pregnancy windows is unclear. However, it might be explained by that fact that people are more likely to change their behavior in response to cold temperature compared with warm temperature. A survey of four large U.S. cities suggested that most participants reported they merely avoided the outdoors despite 90% coverage of heat warning, and over a third of them reported energy cost was an issue for air conditioning use (Sheridan 2007). Similarly, pregnant women may be more likely to use a heater during cold season than air conditioning during warm season, which may partially explain the consistent association between hot temperature and early delivery, but the lack thereof for cold.

According to the National Oceanic and Atmospheric Administration, global temperature has been increasing since the beginning of the 20th century, and the rate of increase has become faster during the last few decades, making 2015 the hottest year on record (NOAA 2011). On the same note, average temperature in the United States has increased > 2°F (> 1°C) over the past 50 years, and it is projected to further increase (Karl et al. 2009). This means that the frequency, duration, and severity of extreme heat events will increase while temperature distribution shifts to the right. If each 5°F increase is associated with a 12–16% increase in risk of early delivery in the warm season, the public health impact is significant given that exposure to ambient temperature is ubiquitous.

This study has some limitations. Approximately 10–30% of women may have relocated during pregnancy (Bell and Belanger 2012). The lack of data on residential history led us to assume that our participants lived and spent time within their hospital referral region. The averaging of temperature across referral regions likely resulted in reduced spatial variation, which may have lowered our power to detect a difference but it is unlikely to explain the significant associations we observed. Further, according to a review of 14 relevant studies, women who relocate during pregnancy typically move within < 10 km (Bell and Belanger 2012) suggesting the use of hospital referral region as a proxy for residence and local mobility may not have seriously affected our findings. One may argue that with a large sample size, our study may be subject to significant findings for small effect size. However, high temperature exposure is ubiquitous, when these results are projected onto large populations, they can have important public health implications. Some babies were born before 28 weeks (*n* = 2,366); therefore, their exposure for weeks 22–28 was averaged for only the weeks in that period before delivery, which could increase the variability in their measurement. Although we adjusted for month of conception and humidity in the main analyses, as well as air pollution in a sensitivity analysis, it is possible that the effects of other seasonally varying risk factors such as influenza infection might remain. However, a recent review (Carolan-Olah and Frankowska 2014) suggests that the relationship between season and preterm birth is unclear, with several studies supporting an increased prevalence in each season. Last, we caution readers that “cold” and “hot” in the chronic exposure windows studied are not universal and refer to the relative temperature extremes in each local area.

The study has several important strengths. The use of site-specific temperature distributions to define extreme temperatures allowed us to account for regional acclimation, a critical concept often overlooked. Meanwhile, specific absolute temperature differences in risk were evaluated using a case-crossover model, which allowed us to adjust for time-invariant confounders. The *Eunice Kennedy Shriver* National Institute of Child Health and Human Development has recommended that special attention should be given to early term births because these infants are also at risk for poor outcomes compared with full-term births (Spong 2013). Although most existing studies typically ignored this group by defining preterm birth at the cut-off of 37 weeks, we are among the first to report increased risk associated with ambient temperature for early term births (Auger et al. 2014). In addition, our study included a large sample size across the United States, which contributes to high generalizability. Last, the consistent findings from multiple sensitivity analyses ensured that our results were robust. Even with some variation, the site-specific findings tend to be consistent with our overall findings that extreme ambient temperature relative to usual environment is important. In addition, we were able to incorporate air pollutant exposures in each pregnancy window to address potential confounding by other ambient exposures.

## Conclusion

In this large U.S. obstetric cohort, we found evidence suggesting that exposures to extreme temperatures during early pregnancy (weeks 1–7) and during the week preceding delivery may be associated with higher risk of early delivery. In addition, chronic whole-pregnancy exposure to hot temperature may also increase the risk for delivery between weeks 34 and 38. Given the recent increase in population risk factors for preterm birth (e.g., maternal age, obesity, gestational complications) and the world-wide concerns related to global warming, our findings highlight the need for awareness among health professionals, policy makers, and women of reproductive age; effective intervention to minimize exposure of pregnant women to extreme temperature; and more research effort on the potential effects of extreme temperatures on adverse birth outcomes.

## Supplemental Material

(205 KB) PDF
