# Supplementary material for: Ambient Temperature and Early Delivery of Singleton Pregnancies
Source: Environ Health Perspect. 2016 Aug 31;125(3):453–9. doi: 10.1289/EHP97 (PMC5332199; doi:10.1289/EHP97)
Supplement: (205 KB) PDF [file EHP97.s001.acco.pdf]

Environ Health Perspect
DOI: 10.1289/EHP97

Note to readers with disabilities: *EHP* strives to ensure that all journal content is accessible to all readers. However, some figures and Supplemental Material published in *EHP* articles may not conform to 508 standards due to the complexity of the information being presented. If you need assistance accessing journal content, please contact ehp508@niehs.nih.gov. Our staff will work with you to assess and meet your accessibility needs within 3 working days.

Supplemental Material

Ambient Temperature and Early Delivery of Singleton Pregnancies

Sandie Ha, Danping Liu, Yeyi Zhu, Sung Soo Kim, Seth Sherman, and Pauline Mendola

Table of Contents

Table S1 Distribution of temperature categories by early delivery status

Table S2 Distributions of site-specific temperature that were used to categorize temperature categories

Table S3 Adjusted relative risk of preterm birth associated with average daily ambient temperature by pregnancy windows

Table S4 Adjusted relative risk of spontaneous preterm birth associated with extreme ambient temperature by pregnancy window

Table S5 Adjusted relative risk of preterm birth associated with average daily ambient temperature by pregnancy windows and study site

Table S6 Adjusted relative risk of preterm birth associated with extreme whole pregnancy temperature up to week of delivery

Table S1. Distribution of temperature categories by early delivery status.

	Early preterm birth (<34 weeks)		Late preterm birth (34-36 weeks)		Early term birth (37-38 weeks)		Full term birth (≥39 weeks)	
	n	%	n	%	n	%	n	%
Chronic exposure[a]								
Preconception								
Cold	951	10.9	1,636	9.4	6,424	9.7	13,377	10.2
Mild	7,008	79.9	13,956	80.4	53,441	80.3	104,233	79.8
Hot	808	9.2	1,771	10.2	6,715	10.1	13,055	10.0
Weeks 1-7								
Cold	1,043	11.9	1,952	11.2	6,876	10.3	12,491	9.6
Mild	6,818	77.8	13,571	78.2	53,028	79.7	105,236	80.5
Hot	906	10.3	1,840	10.6	6,676	10.0	12,938	9.9
Weeks 8-14								
Cold	872	10.0	1,632	9.4	6,510	9.8	13,289	10.2
Mild	7,090	80.9	14,047	80.9	53,288	80.0	104,278	79.8
Hot	805	9.2	1,684	9.7	6,782	10.2	13,098	10.0
Weeks 15-21								
Cold	839	9.6	1,608	9.3	6,501	9.8	13,312	10.2
Mild	7,012	80.0	14,002	80.6	53,442	80.3	104,315	79.8
Hot	916	10.5	1,753	10.1	6,637	10.0	13,038	10.0
Weeks 22-28								
Cold	845	9.6	1,753	10.1	6,511	9.8	13,237	10.1
Mild	6,884	78.5	13,804	79.5	53,350	80.1	104,693	80.1
Hot	1,038	11.8	1,806	10.4	6,719	10.1	12,735	9.8
Acute exposure[b]								
Cold season (Oct-Apr)	48.9	17.2	47.8	16.3	47.0	15.9	47.7[c]	16.4
Warm season (May-Sept)	71.2	9.5	70.6	9.1	70.4	8.8	70.0[c]	9.6

[a]The distribution is presented as frequency (percent). Cold, mild and hot were defined as <10th, 10-90th, and >90th percentile of the distribution of temperature by site.
[b]The distribution is presented as mean (standard deviation) expressed in ˚F. Estimates are based on 87,832 first preterm births.
[c]Season-specific average daily temperature during control periods.

Table S2. Distribution of site-specific absolute temperature that was used to categorize temperature categories.

Pregnancy window	Site	Daily average temperature distribution (˚F)						
		Min	P5	P10	P50	P90	P95	Max
Preconception	1-Baystate Medical Center, Massachusetts	19.9	23.6	27.4	50.4	66.7	67.8	68.9
	2-Cedars-Sinai Medical Center, California	54.6	55.7	56.3	64.7	71.8	73.2	75.6
	3-Christiana Care Health System, Delaware	33.4	35.7	38.0	57.7	75.6	76.5	77.0
	4-Indiana University - Clarian Health, Indiana	31.1	34.2	35.0	56.7	71.6	72.5	73.6
	5-Intermountain HealthCare, Utah	24.4	29.1	31.3	52.0	67.5	68.9	72.0
	6-Maimonides Medical Center, New York	27.8	31.5	34.4	56.8	73.9	74.8	75.7
	7-MedStar Health, Maryland	32.2	35.6	37.8	57.9	73.5	74.3	76.0
	8-MetroHealth Medical Center, Ohio	28.9	30.6	32.0	50.7	68.5	69.4	70.7
	9-Summa Health System, Ohio	26.4	30.1	32.5	53.6	67.9	68.7	70.5
	10-University of Illinois at Chicago, Illinois	26.0	29.0	31.2	47.7	66.5	68.3	70.9
	11-University of Miami, Florida	68.7	69.6	70.1	77.1	83.3	83.5	84.3
	12-University of Texas, Texas	55.2	57.0	58.7	72.7	82.2	82.5	83.6
Weeks 1-7	1-Baystate Medical Center, Massachusetts	14.1	20.5	25.4	48.7	67.4	68.7	70.6
	2-Cedars-Sinai Medical Center, California	44.6	46.7	47.9	60.5	74.7	76.9	79.4
	3-Christiana Care Health System, Delaware	28.9	33.1	37.1	53.9	75.8	77.4	77.9
	4-Indiana University - Clarian Health, Indiana	24.3	31.3	33.1	51.0	71.1	73.1	74.0
	5-Intermountain HealthCare, Utah	19.6	26.0	27.8	50.9	70.6	72.0	77.1
	6-Maimonides Medical Center, New York	23.4	29.7	34.0	56.1	75.0	76.7	77.5
	7-MedStar Health, Maryland	28.9	34.5	37.7	56.9	73.2	74.8	76.6
	8-MetroHealth Medical Center, Ohio	22.0	26.9	31.6	47.9	69.7	70.6	71.7
	9-Summa Health System, Ohio	22.2	27.7	31.1	52.4	68.7	70.7	72.3
	10-University of Illinois at Chicago, Illinois	22.8	27.1	29.6	45.6	68.4	70.6	72.7
	11-University of Miami, Florida	67.9	69.3	69.9	76.4	83.4	83.7	84.2
	12-University of Texas, Texas	51.0	53.2	54.5	69.4	80.5	81.1	82.6
Weeks 8-14	1-Baystate Medical Center, Massachusetts	14.4	19.7	23.0	46.5	67.3	68.6	70.6
	2-Cedars-Sinai Medical Center, California	44.6	46.6	47.6	58.1	74.4	76.9	79.3
	3-Christiana Care Health System, Delaware	29.1	32.3	34.4	54.4	75.9	77.5	77.9
	4-Indiana University - Clarian Health, Indiana	24.2	29.2	31.8	49.0	71.6	73.2	73.9
	5-Intermountain HealthCare, Utah	19.6	25.0	27.4	45.7	70.3	71.9	77.0
	6-Maimonides Medical Center, New York	23.7	29.0	31.9	54.3	75.0	76.7	77.5
	7-MedStar Health, Maryland	29.1	33.0	36.4	55.5	73.3	74.8	76.6
	8-MetroHealth Medical Center, Ohio	22.1	26.7	31.8	48.3	67.7	70.1	71.1
	9-Summa Health System, Ohio	21.8	25.6	28.6	49.6	68.6	70.7	72.2
	10-University of Illinois at Chicago, Illinois	22.8	25.8	28.6	44.5	68.5	70.5	72.7
	11-University of Miami, Florida	67.9	69.2	69.8	76.7	83.4	83.7	84.2
	12-University of Texas, Texas	51.1	53.0	54.3	68.9	80.6	81.0	82.6
Weeks 15-21	1-Baystate Medical Center, Massachusetts	14.4	19.9	23.2	45.3	66.9	68.6	70.6
	2-Cedars-Sinai Medical Center, California	44.6	46.7	47.7	57.2	74.3	76.8	79.3
	3-Christiana Care Health System, Delaware	29.1	34.5	37.5	55.8	75.6	77.4	77.9
	4-Indiana University - Clarian Health, Indiana	24.2	29.8	32.5	50.5	72.5	73.3	73.9
	5-Intermountain HealthCare, Utah	19.6	24.5	27.2	44.1	69.0	71.6	79.5
	6-Maimonides Medical Center, New York	23.7	29.0	31.9	52.8	74.5	76.4	77.5
	7-MedStar Health, Maryland	29.1	32.9	36.4	54.0	73.3	74.8	76.6
	8-MetroHealth Medical Center, Ohio	22.1	25.7	31.6	48.5	68.5	69.9	71.0
	9-Summa Health System, Ohio	21.8	25.8	28.9	47.6	68.5	70.7	72.2
	10-University of Illinois at Chicago, Illinois	22.8	25.8	28.8	44.5	68.2	70.5	72.7

	11-University of Miami, Florida	67.9	69.3	70.0	77.1	83.5	83.8	84.2
	12-University of Texas, Texas	51.1	53.1	54.5	68.9	80.5	80.9	82.2
Weeks 22-28	1-Baystate Medical Center, Massachusetts	14.4	19.9	23.1	45.7	66.9	68.6	71.3
	2-Cedars-Sinai Medical Center, California	43.5	46.6	47.7	57.7	74.4	76.9	80.5
	3-Christiana Care Health System, Delaware	29.7	37.1	38.0	58.0	75.8	77.4	78.7
	4-Indiana University - Clarian Health, Indiana	24.2	30.7	33.5	55.6	72.5	73.3	76.1
	5-Intermountain HealthCare, Utah	19.0	24.8	27.4	44.3	69.3	71.7	79.5
	6-Maimonides Medical Center, New York	23.7	29.0	31.9	53.8	74.3	76.1	77.7
	7-MedStar Health, Maryland	26.4	33.0	36.5	54.2	72.9	74.8	76.8
	8-MetroHealth Medical Center, Ohio	18.3	26.9	31.8	51.1	68.4	69.5	72.7
	9-Summa Health System, Ohio	21.8	25.8	29.0	48.2	68.8	70.7	73.0
	10-University of Illinois at Chicago, Illinois	20.1	26.1	29.2	45.6	68.4	70.6	73.4
	11-University of Miami, Florida	66.9	69.4	70.1	77.4	83.6	83.8	84.6
	12-University of Texas, Texas	49.6	53.1	54.7	69.6	80.5	81.0	82.4

Table S3. Adjusted[a] relative risk of preterm birth associated with extreme ambient temperature by pregnancy windows.

Pregnancy windows	Early preterm birth (<34 weeks)		Late preterm birth (34-36 weeks)		Early term birth (37-38 weeks)	
	RR	(95% CI)	RR	(95% CI)	RR	(95% CI)
Cold						
3 months preconception	1.00	(0.92, 1.09)	**0.92**	(**0.86, 0.98**)	**0.95**	(**0.92, 0.97**)
Weeks 1-7	**1.20**	(**1.11, 1.30**)	**1.09**	(**1.04, 1.15**)	**1.03**	(**1.00, 1.05**)
Weeks 8-14	0.97	(0.90, 1.05)	**0.92**	(**0.87, 0.98**)	**0.95**	(**0.93, 0.98**)
Weeks 15-21	1.00	(0.92, 1.09)	0.94	(0.89, 1.00)	0.97	(0.95, 1.00)
Weeks 22-28	0.94	(0.86, 1.03)	0.97	(0.92, 1.03)	**0.96**	(**0.93, 0.98**)
Hot						
3 months preconception	1.01	(0.92, 1.10)	**1.09**	(**1.02, 1.16**)	1.03	(1.00, 1.05)
Weeks 1-7	**1.11**	(**1.01, 1.21**)	1.05	(0.99, 1.11)	**1.04**	(**1.02, 1.07**)
Weeks 8-14	1.06	(0.96, 1.17)	1.01	(0.94, 1.08)	**1.04**	(**1.01, 1.07**)
Weeks 15-21	**1.18**	(**1.07, 1.29**)	**1.18**	(**1.11, 1.27**)	**1.04**	(**1.01, 1.07**)
Weeks 22-28	1.08	(0.98, 1.19)	1.00	(0.93, 1.07)	1.03	(0.99, 1.06)

Bold face indicates statistical significance at alpha<0.05

Abbreviations: aRR, adjusted relative risk; CI, confidence interval

[a]Model adjusted for all covariates in Table 1, humidity, and study site.

Table S4. Adjusted[a] relative risk of spontaneous preterm birth associated with extreme ambient temperature by pregnancy window.

Pregnancy windows	Early preterm birth (<34 weeks)		Late preterm birth (34-36 weeks)		Early term birth (37-38 weeks)	
	RR	(95% CI)	RR	(95% CI)	RR	(95% CI)
Cold						
3 months preconception	1.06	(0.96, 1.16)	**0.87**	(**0.81, 0.94**)	**0.93**	(**0.90, 0.96**)
Weeks 1-7	**1.21**	(**1.09, 1.33**)	**1.10**	(**1.03, 1.18**)	1.02	(0.99, 1.05)
Weeks 8-14	0.97	(0.88, 1.07)	0.93	(0.87, 1.00)	0.97	(0.94, 1.00)
Weeks 15-21	1.01	(0.91, 1.11)	**0.92**	(**0.85, 0.98**)	0.97	(0.94, 1.00)
Weeks 22-28	0.97	(0.87, 1.07)	0.94	(0.88, 1.01)	0.97	(0.94, 1.00)
Hot						
3 months preconception	0.93	(0.84, 1.03)	1.04	(0.97, 1.11)	**1.03**	(**1.00, 1.06**)
Weeks 1-7	**1.11**	(**1.00, 1.24**)	**1.11**	(**1.03, 1.19**)	**1.04**	(**1.01, 1.08**)
Weeks 8-14	0.98	(0.88, 1.08)	1.01	(0.94, 1.09)	1.02	(0.99, 1.06)
Weeks 15-21	**1.22**	(**1.10, 1.37**)	**1.15**	(**1.07, 1.24**)	1.02	(0.99, 1.05)
Weeks 22-28	**1.15**	(**1.04, 1.27**)	0.99	(0.92, 1.06)	0.99	(0.96, 1.03)

Bold face indicates statistical significance at alpha<0.05

Abbreviations: aRR, adjusted relative risk; CI, confidence interval

[a]Model adjusted for all covariates in Table 1, humidity, and study site.

Table S5. Relative risk of preterm birth associated with extreme ambient temperature by pregnancy windows and site.

Site	Temperature	Pregnancy windows	Early preterm birth (<34 weeks)	Late preterm birth (34-36 weeks)	Early term birth (37-38 weeks)
			RR (95% CI)	RR (95% CI)	RR (95% CI)
1-Baystate Medical Center, Massachusetts	Cold	3 months preconception	1.06 (0.78, 1.43)	1.05 (0.85, 1.31)	1.04 (0.94, 1.14)
		Weeks 1-7	**1.63 (1.18, 2.24)**	1.17 (0.95, 1.44)	1.05 (0.96, 1.16)
		Weeks 8-14	1.09 (0.81, 1.45)	0.99 (0.80, 1.21)	0.99 (0.90, 1.09)
		Weeks 15-21	1.09 (0.81, 1.46)	0.91 (0.72, 1.14)	0.98 (0.89, 1.08)
		Weeks 22-28	0.83 (0.62, 1.11)	0.92 (0.74, 1.15)	1.01 (0.92, 1.11)
	Hot	3 months preconception	0.79 (0.57, 1.10)	1.00 (0.78, 1.28)	0.99 (0.89, 1.10)
		Weeks 1-7	1.37 (0.98, 1.92)	1.17 (0.92, 1.48)	1.07 (0.96, 1.19)
		Weeks 8-14	1.06 (0.76, 1.47)	1.16 (0.92, 1.45)	1.01 (0.91, 1.13)
		Weeks 15-21	0.83 (0.59, 1.18)	0.96 (0.73, 1.25)	0.93 (0.82, 1.04)
		Weeks 22-28	0.80 (0.55, 1.16)	0.93 (0.72, 1.21)	0.94 (0.84, 1.05)
2-Cedars-Sinai Medical Center, California	Cold	3 months preconception	1.35 (0.87, 2.10)	1.16 (0.91, 1.49)	1.04 (0.95, 1.15)
		Weeks 1-7	1.21 (0.79, 1.85)	**1.70 (1.33, 2.17)**	**1.16 (1.06, 1.27)**
		Weeks 8-14	0.84 (0.58, 1.23)	1.04 (0.83, 1.30)	0.90 (0.82, 1.00)
		Weeks 15-21	0.92 (0.63, 1.35)	0.98 (0.78, 1.22)	1.02 (0.93, 1.12)
		Weeks 22-28	0.86 (0.57, 1.29)	1.10 (0.88, 1.37)	1.01 (0.92, 1.11)
	Hot	3 months preconception	0.78 (0.51, 1.19)	0.89 (0.69, 1.14)	1.00 (0.90, 1.10)
		Weeks 1-7	1.48 (0.91, 2.40)	0.91 (0.71, 1.18)	1.00 (0.90, 1.13)
		Weeks 8-14	1.53 (0.95, 2.45)	1.13 (0.85, 1.52)	1.07 (0.96, 1.20)
		Weeks 15-21	1.33 (0.80, 2.22)	1.37 (0.99, 1.89)	1.09 (0.96, 1.24)
		Weeks 22-28	**0.45 (0.21, 0.98)**	0.42 (0.28, 0.64)	0.85 (0.71, 1.02)
3-Christiana Care Health System, Delaware	Cold	3 months preconception	0.68 (0.42, 1.12)	1.24 (0.91, 1.69)	0.93 (0.81, 1.06)
		Weeks 1-7	1.11 (0.73, 1.68)	1.21 (0.95, 1.55)	1.09 (0.98, 1.21)
		Weeks 8-14	**0.30 (0.16, 0.54)**	0.83 (0.62, 1.11)	0.93 (0.83, 1.04)
		Weeks 15-21	1.03 (0.67, 1.58)	**0.64 (0.46, 0.88)**	0.97 (0.86, 1.09)
		Weeks 22-28	1.04 (0.71, 1.53)	0.84 (0.66, 1.07)	**0.88 (0.78, 0.98)**
	Hot	3 months preconception	1.10 (0.62, 1.94)	0.85 (0.58, 1.25)	1.02 (0.86, 1.22)
		Weeks 1-7	1.37 (0.84, 2.22)	**0.73 (0.54, 0.99)**	1.05 (0.93, 1.20)
		Weeks 8-14	0.53 (0.27, 1.03)	1.08 (0.72, 1.60)	0.98 (0.83, 1.15)
		Weeks 15-21	0.93 (0.59, 1.46)	1.14 (0.84, 1.53)	1.05 (0.92, 1.20)
		Weeks 22-28	0.63 (0.37, 1.08)	0.74 (0.55, 1.00)	1.05 (0.91, 1.21)
4-Indiana	Cold	3 months preconception	0.66 (0.36, 1.21)	1.31 (0.87, 1.98)	0.92 (0.75, 1.11)

University - Clarian Health, Indiana		Weeks 1-7	1.27 (0.64, 2.50)	1.13 (0.82, 1.55)	1.05 (0.90, 1.22)
		Weeks 8-14	0.64 (0.31, 1.33)	**0.66 (0.48, 0.91)**	**0.80 (0.69, 0.94)**
		Weeks 15-21	1.42 (0.75, 2.69)	0.89 (0.60, 1.33)	0.95 (0.80, 1.13)
		Weeks 22-28	0.46 (0.19, 1.11)	0.70 (0.46, 1.08)	0.88 (0.75, 1.05)
	Hot	3 months preconception	1.80 (0.91, 3.55)	1.29 (0.86, 1.95)	1.01 (0.86, 1.19)
		Weeks 1-7	1.47 (0.54, 4.00)	0.77 (0.47, 1.28)	**1.34 (1.03, 1.75)**
		Weeks 8-14	1.33 (0.57, 3.13)	0.92 (0.51, 1.64)	1.10 (0.87, 1.39)
		Weeks 15-21	0.67 (0.36, 1.24)	1.27 (0.82, 1.97)	1.10 (0.91, 1.34)
		Weeks 22-28	1.34 (0.70, 2.58)	0.82 (0.56, 1.21)	0.98 (0.80, 1.19)
5-Intermountain HealthCare, Utah	Cold	3 months preconception	1.16 (0.84, 1.60)	0.84 (0.71, 1.00)	**0.88 (0.82, 0.94)**
		Weeks 1-7	1.01 (0.82, 1.25)	1.08 (0.96, 1.22)	1.04 (0.98, 1.09)
		Weeks 8-14	0.93 (0.74, 1.18)	0.87 (0.76, 1.00)	0.95 (0.90, 1.00)
		Weeks 15-21	0.90 (0.71, 1.15)	1.00 (0.87, 1.14)	0.95 (0.90, 1.00)
		Weeks 22-28	1.02 (0.78, 1.34)	1.02 (0.89, 1.17)	**0.92 (0.87, 0.97)**
	Hot	3 months preconception	1.11 (0.85, 1.46)	1.10 (0.94, 1.28)	1.04 (0.98, 1.10)
		Weeks 1-7	**1.58 (1.19, 2.09)**	**1.26 (1.09, 1.46)**	**1.10 (1.04, 1.17)**
		Weeks 8-14	**1.44 (1.02, 2.04)**	**1.27 (1.06, 1.52)**	**1.12 (1.05, 1.20)**
		Weeks 15-21	**1.73 (1.23, 2.42)**	**1.29 (1.06, 1.58)**	1.06 (0.97, 1.15)
		Weeks 22-28	**1.81 (1.17, 2.80)**	**1.39 (1.12, 1.73)**	**1.14 (1.04, 1.25)**
6-Maimonides Medical Center, New York	Cold	3 months preconception	0.78 (0.50, 1.23)	**0.59 (0.44, 0.79)**	0.92 (0.82, 1.03)
		Weeks 1-7	0.86 (0.55, 1.34)	1.03 (0.80, 1.33)	0.95 (0.86, 1.05)
		Weeks 8-14	1.22 (0.77, 1.91)	0.92 (0.72, 1.18)	0.96 (0.87, 1.06)
		Weeks 15-21	0.74 (0.43, 1.25)	1.03 (0.78, 1.36)	1.00 (0.90, 1.12)
		Weeks 22-28	1.22 (0.77, 1.93)	1.19 (0.91, 1.54)	1.07 (0.96, 1.18)
	Hot	3 months preconception	1.23 (0.75, 2.03)	0.96 (0.71, 1.31)	1.04 (0.92, 1.17)
		Weeks 1-7	0.87 (0.53, 1.41)	1.20 (0.88, 1.62)	1.00 (0.89, 1.13)
		Weeks 8-14	0.83 (0.47, 1.47)	**0.74 (0.56, 0.99)**	1.05 (0.93, 1.17)
		Weeks 15-21	0.72 (0.41, 1.25)	1.05 (0.73, 1.49)	0.95 (0.84, 1.09)
		Weeks 22-28	0.68 (0.35, 1.30)	0.80 (0.60, 1.08)	1.00 (0.88, 1.14)
7-MedStar Health, Maryland	Cold	3 months preconception	**0.42 (0.31, 0.57)**	**0.70 (0.57, 0.86)**	0.95 (0.87, 1.03)
		Weeks 1-7	**1.55 (1.25, 1.92)**	**1.38 (1.18, 1.62)**	**1.11 (1.03, 1.19)**
		Weeks 8-14	0.95 (0.73, 1.23)	0.96 (0.81, 1.15)	0.97 (0.90, 1.05)
		Weeks 15-21	0.85 (0.63, 1.14)	1.00 (0.83, 1.20)	0.94 (0.87, 1.03)
		Weeks 22-28	1.08 (0.83, 1.40)	1.05 (0.89, 1.24)	0.94 (0.87, 1.01)
	Hot	3 months preconception	1.05 (0.78, 1.43)	**1.36 (1.13, 1.64)**	**1.12 (1.02, 1.22)**
		Weeks 1-7	1.14 (0.86, 1.50)	1.19 (0.99, 1.43)	**1.13 (1.03, 1.23)**

		Weeks 8-14	1.01 (0.76, 1.34)	1.04 (0.85, 1.27)	**1.08 (1.00, 1.18)**
		Weeks 15-21	**2.21 (1.62, 3.02)**	1.18 (0.96, 1.45)	1.08 (0.99, 1.18)
		Weeks 22-28	0.81 (0.59, 1.13)	**0.77 (0.62, 0.96)**	0.97 (0.87, 1.08)
8-MetroHealth Medical Center, Ohio	Cold	3 months preconception	0.89 (0.56, 1.41)	**0.63 (0.44, 0.91)**	0.96 (0.79, 1.16)
		Weeks 1-7	1.02 (0.68, 1.54)	1.04 (0.78, 1.37)	1.02 (0.87, 1.18)
		Weeks 8-14	0.88 (0.57, 1.35)	0.95 (0.70, 1.28)	0.96 (0.82, 1.12)
		Weeks 15-21	1.19 (0.77, 1.85)	1.15 (0.86, 1.53)	1.13 (0.97, 1.33)
		Weeks 22-28	1.16 (0.75, 1.78)	0.88 (0.65, 1.20)	1.13 (0.97, 1.32)
	Hot	3 months preconception	0.91 (0.57, 1.45)	1.14 (0.82, 1.58)	0.98 (0.82, 1.16)
		Weeks 1-7	**0.29 (0.17, 0.49)**	1.08 (0.74, 1.56)	**0.78 (0.64, 0.94)**
		Weeks 8-14	0.56 (0.26, 1.20)	**0.37 (0.21, 0.63)**	**0.71 (0.57, 0.89)**
		Weeks 15-21	1.22 (0.74, 2.01)	0.97 (0.67, 1.39)	0.96 (0.79, 1.18)
		Weeks 22-28	1.45 (0.87, 2.42)	0.77 (0.54, 1.10)	1.05 (0.86, 1.29)
9-Summa Health System, Ohio	Cold	3 months preconception	1.25 (0.78, 2.00)	0.75 (0.51, 1.11)	**0.76 (0.64, 0.91)**
		Weeks 1-7	1.14 (0.73, 1.79)	1.35 (0.98, 1.87)	0.99 (0.85, 1.15)
		Weeks 8-14	1.30 (0.81, 2.09)	1.05 (0.75, 1.46)	1.00 (0.86, 1.15)
		Weeks 15-21	1.07 (0.62, 1.84)	0.72 (0.50, 1.03)	1.11 (0.94, 1.31)
		Weeks 22-28	0.94 (0.55, 1.61)	1.05 (0.76, 1.45)	1.02 (0.88, 1.19)
	Hot	3 months preconception	0.97 (0.62, 1.51)	1.08 (0.81, 1.45)	**0.82 (0.71, 0.94)**
		Weeks 1-7	0.93 (0.59, 1.46)	0.83 (0.62, 1.10)	0.87 (0.75, 1.00)
		Weeks 8-14	**2.54 (1.52, 4.26)**	1.03 (0.72, 1.47)	1.07 (0.92, 1.25)
		Weeks 15-21	0.85 (0.50, 1.44)	**1.60 (1.08, 2.35)**	1.15 (0.98, 1.36)
		Weeks 22-28	0.84 (0.42, 1.70)	**0.62 (0.42, 0.91)**	0.90 (0.72, 1.12)
10-University of Illinois at Chicago, Illinois	Cold	3 months preconception	1.16 (0.91, 1.48)	1.07 (0.85, 1.35)	1.00 (0.88, 1.13)
		Weeks 1-7	**1.40 (1.08, 1.81)**	1.12 (0.88, 1.42)	1.07 (0.94, 1.21)
		Weeks 8-14	1.03 (0.80, 1.31)	0.96 (0.76, 1.21)	0.96 (0.84, 1.08)
		Weeks 15-21	**1.64 (1.21, 2.23)**	1.01 (0.79, 1.30)	1.01 (0.88, 1.16)
		Weeks 22-28	0.82 (0.61, 1.11)	0.87 (0.68, 1.11)	0.88 (0.77, 1.00)
	Hot	3 months preconception	1.05 (0.77, 1.43)	1.12 (0.85, 1.46)	1.07 (0.93, 1.23)
		Weeks 1-7	0.83 (0.62, 1.10)	0.89 (0.70, 1.13)	0.99 (0.87, 1.13)
		Weeks 8-14	0.82 (0.61, 1.10)	0.93 (0.70, 1.24)	0.99 (0.85, 1.15)
		Weeks 15-21	0.91 (0.68, 1.20)	0.88 (0.66, 1.17)	0.90 (0.78, 1.04)
		Weeks 22-28	1.10 (0.83, 1.46)	1.07 (0.82, 1.38)	1.11 (0.97, 1.27)
11-University of Miami, Florida	Cold	3 months preconception	1.05 (0.80, 1.37)	0.89 (0.72, 1.11)	**0.86 (0.77, 0.96)**
		Weeks 1-7	0.94 (0.73, 1.21)	1.12 (0.90, 1.38)	1.01 (0.90, 1.12)
		Weeks 8-14	1.02 (0.79, 1.31)	0.84 (0.68, 1.05)	0.90 (0.81, 1.01)

		Weeks 15-21	1.07 (0.79, 1.46)	1.01 (0.79, 1.28)	0.95 (0.84, 1.08)
		Weeks 22-28	1.17 (0.89, 1.53)	1.01 (0.83, 1.24)	1.03 (0.93, 1.15)
	Hot	3 months preconception	1.19 (0.89, 1.57)	1.09 (0.86, 1.38)	0.99 (0.88, 1.12)
		Weeks 1-7	0.96 (0.75, 1.24)	1.12 (0.91, 1.38)	1.00 (0.90, 1.12)
		Weeks 8-14	1.13 (0.86, 1.47)	1.13 (0.92, 1.38)	1.04 (0.93, 1.16)
		Weeks 15-21	**1.36 (1.05, 1.75)**	**1.48 (1.20, 1.81)**	**1.23 (1.10, 1.37)**
		Weeks 22-28	1.04 (0.77, 1.40)	1.26 (0.99, 1.61)	1.02 (0.91, 1.14)
12-University of Texas, Texas	Cold	3 months preconception	1.08 (0.88, 1.33)	1.02 (0.87, 1.20)	0.96 (0.90, 1.03)
		Weeks 1-7	1.12 (0.92, 1.36)	0.87 (0.75, 1.01)	0.98 (0.92, 1.04)
		Weeks 8-14	1.05 (0.86, 1.28)	**0.81 (0.69, 0.94)**	**0.91 (0.86, 0.98)**
		Weeks 15-21	1.00 (0.81, 1.22)	0.97 (0.83, 1.13)	0.96 (0.90, 1.02)
		Weeks 22-28	1.05 (0.84, 1.33)	0.88 (0.75, 1.03)	0.95 (0.88, 1.01)
	Hot	3 months preconception	1.07 (0.85, 1.35)	1.03 (0.86, 1.23)	0.99 (0.92, 1.07)
		Weeks 1-7	1.12 (0.87, 1.44)	0.97 (0.81, 1.15)	0.99 (0.92, 1.06)
		Weeks 8-14	0.89 (0.67, 1.18)	0.88 (0.73, 1.07)	0.95 (0.89, 1.03)
		Weeks 15-21	0.99 (0.80, 1.23)	**1.21 (1.02, 1.43)**	**1.07 (1.00, 1.14)**
		Weeks 22-28	1.13 (0.90, 1.42)	0.92 (0.77, 1.10)	0.99 (0.92, 1.07)

Bold face indicates statistical significance at alpha<0.05. Models were adjusted for month of conception.

Abbreviations: aRR, adjusted relative risk; CI, confidence interval

Table S6. Adjusted relative risk of preterm birth associated with extreme whole pregnancy temperature up to week of delivery.

	RR[a] (95% CI)		n	
Week of delivery	Cold	Hot	Delivered	Ongoing pregnancies
23	1.02 (0.64, 1.62)	1.32 (0.84, 2.08)	416	222,959
24	0.98 (0.66, 1.47)	1.37 (0.93, 2.02)	496	222,463
25	1.26 (0.85, 1.87)	1.04 (0.66, 1.63)	464	221,999
26	1.29 (0.90, 1.85)	1.37 (0.89, 2.11)	475	221,524
27	1.01 (0.72, 1.42)	0.88 (0.58, 1.33)	515	221,009
28	0.97 (0.68, 1.39)	1.46 (0.99, 2.14)	576	220,433
29	0.92 (0.65, 1.30)	0.87 (0.61, 1.25)	695	219,738
30	1.17 (0.89, 1.53)	1.28 (0.92, 1.78)	860	218,878
31	0.97 (0.74, 1.28)	0.77 (0.55, 1.08)	1,026	217,852
32	1.00 (0.79, 1.26)	1.10 (0.86, 1.42)	1,395	216,457
33	0.99 (0.81, 1.20)	0.91 (0.73, 1.15)	1,849	214,608
34	0.96 (0.82, 1.12)	**1.21 (1.01, 1.44)**	2,948	211,660
35	1.00 (0.88, 1.13)	1.08 (0.94, 1.24)	4,759	206,901
36	0.99 (0.91, 1.08)	**1.16 (1.06, 1.28)**	9,656	197,245
37	**0.93 (0.88, 0.98)**	**1.08 (1.01, 1.15)**	21,032	176,213
38	**0.94 (0.91, 0.97)**	**1.06 (1.02, 1.10)**	45,548	130,665

Bold face indicates statistical significance at alpha<0.05

Abbreviations: RR, relative risk; CI, confidence interval

[a] Model adjusted for all covariates in Table 1, humidity, and study site.
